# *Massive Open Online Courses* for continuing education for nursing professionals in Peru*

**DOI:** 10.1590/1518-8345.3803.3297

**Published:** 2020-07-01

**Authors:** Guido Bendezu-Quispe, Renatta Quijano-Escate, Akram Hernández-Vásquez, Fiorella Inga-Berrospi, Daniel Flavio Condor

**Affiliations:** 1 Universidad San Ignacio de Loyola, Unidad de Investigación para la Generación y Síntesis de Evidencias en Salud, Lima, Peru.; 2 Universidad Nacional San Luis Gonzaga, Sociedad Científica de Estudiantes de Medicina de Ica - SOCEMI, Ica, Peru.; 3 Universidad San Ignacio de Loyola, Centro de Excelencia en Investigaciones Económicas y Sociales en Salud, Lima, Peru.; 4 Universidad Privada Norbert Wiener, Centro de investigación, Lima, Peru.; 5 Universidad Continental, Lima, Peru.; 6 Universidad Peruana Cayetano Heredia, Facultad de Enfermería, Lima, Peru.

**Keywords:** Education, Nursing, Nurses Instruction, Education, Nursing,Continuing, Specialties, Nursing, Education, Nursing, Graduate, Nurse Specialists, Educação em Enfermagem, Instrução para Enfermeiros, Educação Continuada em Enfermagem, Especialidades de Enfermagem, Educação de Pós-Graduação em Enfermagem, Enfermeiras Especialistas, Educación en Enfermería, Instrucción para Enfermeras, Educación Continua en Enfermería, Especialidades de Enfermería, Educación de Posgrado en Enfermería, Enfermeras Especialistas

## Abstract

to determine the global offer of Massive Open Online Courses (MOOCs) in health and nursing, and to know the characteristics of its content, for continuing education in nursing professionals in Peru.

**Method:**

exploratory study was carried out on the websites: Coursera, edX, FutureLearn, XuetangX and Udacity, Class Central and MOOC List. The courses were classified according to the five nursing areas recognized by the Peruvian College of Nurses (*Colegio de Enfermeros del Perú*, CEP). From each course, data was collected on institution and country of origin, hours per week and total duration in weeks, audio and subtitle language.

**Results:**

a total of 654 courses in this modality are offered in health were found, covering the five areas contemplated by the Peruvian College of Nurses. Fourteen courses were specifically developed for nursing with an average duration of five weeks (3.2 hours per week of activities). Eleven came from Anglo-Saxon institutions, with content in English. Only two courses were offered in Spanish and one in Turkish.

**Conclusion:**

Massive Open Online Courses would be a useful tool for the continuing education of the Peruvian nurse given the wide offer, including some specifically for nurses, in the different areas of nursing. The content of the course is mostly in English. Expanding the range of languages or subtitles would facilitate the participation of a larger audience.

## Objetivo

determinar a oferta global de *Massive Open Online Courses* (MOOCs) em saúde e enfermagem e conhecer as características de seu conteúdo para a educação continuada em profissionais de enfermagem.

**Método:**

estudo exploratório realizado nos sites Coursera, edX, FutureLearn, XuetangX e Udacity, *Class Central* e MOOC *List*. Os cursos foram classificados de acordo com as cinco áreas de enfermagem reconhecidas pelo Colégio Peruano de Enfermeiros (*Colegio de Enfermeros del Perú*, CEP). Para cada curso foram coletadas as variáveis instituição e país de origem, horas semanais e duração total em semanas, idioma do áudio e das legendas.

**Resultados:**

foram encontrados 654 cursos nessa modalidade em saúde, cobrindo as cinco áreas contempladas pelo Colégio Peruano de Enfermeiros. Quatorze cursos foram desenvolvidos especificamente para enfermagem, com duração média de cinco semanas (3,2 horas por semana de atividades). Onze procediam de instituições anglo-saxônicas, com conteúdo em inglês. Apenas dois cursos foram oferecidos em espanhol e um em turco.

**Conclusão:**

*Massive Open Online Courses* é uma ferramenta útil para a educação continuada do enfermeiro peruano, dada a ampla oferta, incluindo alguns especificamente para enfermeiros, nas diferentes áreas da enfermagem. O seu conteúdo é oferecido principalmente em inglês. Expandir a variedade de idiomas ou incluir legendas facilitaria a participação de um público maior.

determinar la oferta global de *Massive Open Online Courses* (MOOCs) en salud y enfermería, y conocer las características de su contenido, para la educación continua de profesionales de enfermería en Perú.

**Método:**

estudio exploratorio se llevó a cabo en sitios web Coursera, edX, FutureLearn, XuetangX y Udacity, *Class Central* y MOOC List. Los cursos se clasificaron según las cinco áreas de enfermería reconocidas por el Colegio de Enfermeros del Perú (CEP). De cada curso se recopilaron datos sobre institución y país de origen, horas por semana y duración total en semanas, idioma de audio y de subtítulos.

**Resultados:**

se encontró un total de 654 cursos en esta modalidad en salud, que abarcaron las cinco áreas contempladas por el Colegio de Enfermeros del Perú. Catorce cursos fueron elaborados específicamente para enfermería con una duración promedio de cinco semanas (3,2 horas semanales de actividades). Once procedían de instituciones anglosajonas, con contenido en inglés. Solo dos se ofrecieron en español y uno en turco.

**Conclusión:**

*Massive Open Online* Courses es una herramienta útil para la educación continua del enfermero peruano dada la amplia oferta, incluyendo algunos específicamente para enfermeros, en las distintas áreas de la enfermería. El contenido de los cursos se ofrecen mayoritariamente en inglés. Ampliar la oferta de idiomas o subtítulos facilitaría la participación de un mayor público.

## Introduction

Health professionals require constant training and the acquisition of new knowledge and skills for their job performance that allows them to respond to the health needs of the population during their work activities^(^ 12 ^-^ 2 ^)^. Nursing is one of the professions that, historically and with the greatest boom in recent decades, has shown interest in developing programs that provide constant professional training^(^ 3 ^)^. Worldwide, despite the availability of in person courses, mostly provided by academic institutions for continuing education in nursing, professionals increasingly find it difficult to take in person courses to continue their training due to various factors, including economic, family and availability ones^(^ 1 ^,^ 4 ^)^. This scenario has generated an increase in the number of participants in part-time courses or courses available on the Internet^(^ 5 ^-^ 6 ^)^, which also facilitate the dissemination of knowledge with greater accessibility and reducing costs, the latter being desirable characteristics for professionals in low and middle income countries^7^^)^.

The continuous certification and reaching higher academic degrees by nursing professionals is associated with an improvement in the clinical outcomes of patients during their professional practice and in their professional performance^(^ 8 ^-^ 9 ^)^. The dizzying emergence of new knowledge and technological advances demands constant updating by nursing professionals. In recent years, there has been a worldwide boom in Massive Open Online Courses (MOOCs), courses considered as a disruptive innovation in education, with a philosophy of democratizing access to knowledge through the offering of courses available to anyone with Internet access and regardless of the time or place from which access, allowing these types of courses to have a global audience. Since 2008, considered the beginning of the appearance of these courses, MOOCs have been developed in various areas of human knowledge, including the area of health^(^ 10 ^)^. The attainment of a MOOC course, against payment or not (depending on the course), can generate the student a certificate of completion of the course useful to verify knowledge or learning^(^ 10 ^)^.

In Peru, in 2002 Law No 27669 - “Law on the Work of the Nurse” was issued. This document indicates that continuing education, training, and permanent improvement are inherent to the work of the nursing professional in Peru, also pointing out that professionals who provide services in less developed areas should receive training according to the needs of their community^(^ 11 ^)^. In this way, the need for continuous training for the nursing health professional is recognized. In addition, the aforementioned law states that, within Comprehensive Nursing Care, made up of five areas, the teaching area has to include the training of nurses and continuing education in nursing^(^ 11 ^)^.

Although online education, including MOOCs, is recognized as an alternative to continuing education for health professionals^(^ 10 ^,^ 12 ^-^ 13 ^)^, the offer and content of MOOCs in health have not been explored, nor specifically for the continuing education of nursing professionals, having only described isolated experiences on the development of nursing MOOCs in the world^14^^-^ 15 ^)^. Therefore, the objective of this study was to determine the global offer of MOOCs in health and specifically for nursing professionals and to know the characteristics of its content, for continuing education in nursing professionals in Peru. It is hoped that the rise of MOOCs can be useful as a tool for continuing education in nursing professionals in Peru.

## Method

A manual search was carried out from March 15 to April 25, 2019 in learning platforms that offer MOOCs including Coursera, edX, FutureLearn, XuetangX and Udacity, to identify and know the characteristics of MOOCs in health or nursing with topics according to the Nursing areas and specialties recognized by the Peruvian College of Nurses (CEP)^(^ 16 ^)^, which were the terms included in the search tools available in the learning platforms. These platforms were used to search for MOOCs in health or nursing since their websites include the widest range of MOOCs and have the largest number of registered users^(^ 17 ^)^, in addition to having been used by previous studies on health MOOCs^10^^,^ 182 ^-^ 19 ^)^. Additionally, a manual search for health MOOCs was carried out on the Class Central (https://www.classcentral.com/) and MOOC List (https://www.mooc-list.com/) websites. Because they are platforms that bring together MOOCs from various platforms (including those previously mentioned and others, such as: Miríada X, Alison, FUN.MOOC, UPV [X], Canvas Network, OpenWHO, Formación Alcalá, Open2Study, among others).

In a first phase, MOOCs in health were identified, classified according to the area of nursing according to the CEP. The CEP promulgated, in 2015, Resolution No. 357-15 CN/CEP, recognizing 5 areas of nursing, including: Professional care (21 specialties); Public health, family and community health (4 specialties); Teaching and research (2 specialties), Organizational and management (2 specialties) and Professional development (03 specialties). To learn more about the areas of nursing and specialty recognized by the CEP, you can review the resolution promulgated^(^ 16 ^)^.

Subsequently, the MOOCS developed specifically for nursing professionals were identified, collecting the following data: institution, country, duration of the course in weeks, and time required *per week* in hours, language and subtitles available for each course. The classification of MOOCs according to area was carried out by two of the authors who carried out the review independently during the month of May 2019. Once the courses were compared by each reviewer, with a previous discard of MOOCs found in duplicate, in case no agreement was found regarding the classification on the area or specialty assigned to the MOOC, a third author evaluated these cases in particular to establish the area and specialty to which the course belonged.

The information obtained on MOOCs in health and nursing was reported in frequencies and percentages for the case of categorical data and using means and ranges for numerical data. The aforementioned calculations were performed using Microsoft Excel 2013 software (Microsoft Corporation, USA).

This work did not require approval by the ethics committee as it was a study of secondary data in the public domain.

## Results

A total of 654 MOOCs in health were found with content related to the five areas covered by the CEP. The area with the highest number of MOOCs in health was Public, Family and Community Health with 35.8% of courses followed by the Professional Care area (26.6%). The professional development area was the one where the least offer of MOOCs courses was found, being only 12 (1.8%) (Table 1).

**Table 1:** General characteristics of MOOCs* on health and nursing, in the world, 2019

Characteristic	Frequency	Percentage (%)
Audience		
Health	640	97.9
Nursing	14	2.1
Area and specialty^†^		
Professional care	174	26.6
Public, family and Community Health	234	35.8
Teaching and research	136	21.0
Organizational and management	97	14.8
Professional development	12	1.8

* MOOCs = Massive Open Online Courses;

† Classification based on Nursing Areas and Specialties of resolution No. 357-15 CN/CEP of the College of Nurses of Peru

Regarding nursing-oriented courses, 14 courses specifically developed for this profession were found. Regarding the origin of the courses, the majority came from Anglo-Saxon countries (11/14), 6/14 being prepared by institutions in the United Kingdom and 4/14 by institutions in the United States of America. Coventry University was the institution with the highest number of MOOCs offered for the nursing professional with 3/14 courses, followed by *Formación Alcalá* with two courses. The other institutions offered one MOOC each (Table 2).

**Table 2:** Characteristics of MOOCs* focused on nursing in online learning platforms, in the World, 2019

Title	Country	Duration^†^	Language (Subtitles)	CEP^‡^ Area
Care Home Nursing: Changing Perceptions	United Kingdom	4 (2)	English	Professional care
Understanding NHS Values: Becoming a Nurse or Midwife	United Kingdom	2 (2)	English	Teaching and research
Preparing for a Career in Nursing	USA	5 (2)	English	Teaching and research
English for Nurses: A Language Learning Community	USA	4 (4)	English	Teaching and research
The Impact of Nursing	United Kingdom	4 (3)	English	Teaching and research
Introduction to Nursing in Healthcare	Australia	4 (2-4)	English	Teaching and research
Rural Health Nursing	USA	8	English	Public, family and community health
İnfertilite Hemşireliği (Infertility Nursing)	Turkey	8 (4-6)	Turkish	Professional care
El recién nascido normal	Spain	(7)	Spanish	Professional care
Proceso de donación de sangre	Spain	(5)	Spanish	Organizational and management
Infection Prevention in Nursing Homes	USA	6	English	Public, family and community health
Could You Be the Next Florence Nightingale?	United Kingdom	6 (2)	English	Public, family and community health
Nursing and Long-term Conditions: An Introduction	United Kingdom	6 (2)	English	Public, family and community health
Learning Theory for Nursing: An Introduction	United Kingdom	3 (2)	English	Teaching and research

* MOOCs = Massive Open Online Courses;

† Duration in weeks. The number of hours per week is indicated in parentheses;

‡ CEP = College of Nurses of Peru;

§ USA = United States of America

Regarding the characteristics of the MOOCs in nursing, these had an average duration of five weeks (range: 2 - 8), with an average of 3.2 hours of activities *per week* (range: 2 - 7). Six of the courses would develop the Teaching and Research area and four the Public, Family and Community Health area. No nursing MOOCs were found in the Professional Development area (Table 2). Regarding the language, eleven of the courses were offered in English, two in Spanish and one in Turkish with their respective transcripts of the audiovisual material in the same languages. The nursing MOOCs found did not have available subtitles of their content in other languages (Table 2).

## Discussion

In this work, we sought to determine the global offer of MOOCs in health or nursing and to know the characteristics of their content. As findings, an important offer of MOOCs in health was found, with courses in each of the areas of nursing according to the classification given by the CEP, and there are also courses offered specifically for the nursing professional. This extensive online educational offer would indicate that health professionals, including nurses in Peru, have, to date, an alternative for training in different areas of their professional work, different from traditional learning systems (in person courses).

More than 600 MOOCs were found available in the health area, this content being potentially useful for the training of the nursing professional. In this regard, this high number of courses would indicate the interest of the academy in the development of MOOCs in the area of health, with a growth in terms of the offer of them compared to 2014, where a review of MOOCs in the health field reported an offer of 98 courses on 12 MOOC platforms^(^ 10 ^)^. The nursing professional requires constant updating of their knowledge, which makes it necessary to develop courses that reduce knowledge gaps in current health scenarios. MOOCs have been described as being useful in the health field, with courses offering focused on topics of current relevance such as mental health^(^ 18 ^)^, training on current global health topics such as emerging infectious diseases^19^^)^, and medical education^20^^)^. Having found MOOCs in all areas of nursing, according to the CEP classification, with courses specifically developed for the nursing professional, would indicate that MOOCs would be a useful tool in the field of nursing.

One of the goals of continuing education for health professionals is to improve professional performance. In this regard, a higher educational level and experience has been described related to lower mortality and better clinical response of patients in the acute area^(^ 21 ^)^. In addition, MOOCS as an alternative available to anyone with Internet access may be an option for groups suffering from professional isolation, such as professionals working in rural and remote areas^(^ 22 ^)^. The offer of online courses would allow nurses to continue their training in a cost-effective way, in a professional setting in which it is increasingly difficult to attend traditionally taught courses (development courses that require physical presence of the student and educational facilitator). In this regard, it has been described that increasingly, nursing professionals take online courses to continue their training and obtain training^(^ 23 ^-^ 25 ^)^. In this study, it was found that some of the areas with the highest number of MOOCs are public health and research. Finding courses in research in the health field is consistent with a global research agenda and interest in learning evidence-based practices in the field of nursing and health in general^(^ 23 ^)^.

Most of the MOOCs in health and nursing are provided in the English language, elaborated mostly by recognized academic institutions from Anglo-Saxon countries^(^ 10 ^)^. This result is in accordance with the general panorama on MOOCs, where 75% of courses on MOOC platforms are offered in that language^(^ 26 ^)^. Therefore, the development of the offer in different languages, or the possibility of offering the content of the courses in more than one language, could favor their use and increase the target population that can benefit from their content, being that most MOOCs are conducted by Anglo-Saxon educational institutions. In general, Latin American institutions have a low participation in the development of MOOCs in health^(^ 27 ^)^, which could explain the low supply of courses in health and nursing in Spanish. This situation may mean that most of the courses taken do not respond to the continuing education or training needs of professionals according to the priorities of the Latin American region, making it necessary to promote courses that address topics of regional interest^(^ 28 ^)^.

The finding that few nursing MOOCs only have transcripts in the source language, with no subtitles available for other languages, indicates that these courses, although they are tools easily accessible with the internet, would not be useful for professionals who do not have knowledge of English or another language used in MOOCs other than their native language. In Peru, since the promulgation of the new University Law (Law No. 30220), obtaining a graduate degree (master’s or doctorate) requires the knowledge of a foreign language by the graduate, recommending the English language^(^ 29 ^)^. This requirement also applies to undergraduate studies in Peru^(^ 29 ^)^. Given that the number of people, including professionals, who have an adequate level of English to be able to take academic courses is still low in Peru^(^ 30 ^)^, finding an offer of MOOCs predominantly in English, with no subtitle options, indicates that a large proportion of the courses offered would not be useful for the professional who wants to train through these online tools. This finding represents an important limitation for the use of this technology in the training of nurses in Peru, and it is necessary to promote language learning from high school, which would allow future graduates greater access to professional updating courses.

Regarding the duration of MOOCs specific to the nursing area, it should be noted that the average hourly load of three hours *per week* for courses of average duration of five hours would be appropriate for nursing professionals since, although it has been described an increase in the number of professionals taking online courses such as MOOCs, one of the main weaknesses of Internet teaching is the low proportion of participants who complete all of these courses, with the dropout rate in participants being low in this type of teaching can be up to six times that evidenced in classroom courses^31^^)^. In this way, a lower hourly load could facilitate the participant to be able to comply with the entire program proposed in a MOOC. In line with the challenges of online teaching, the need for technical support for the development of the courses has been described, the development of tools that allow interaction (in real time or feedback) for the student, since the involvement of the student increases the probability of completion of the course and the still (although less and less important) technical ability of the student on the use of technological devices^(^ 32 ^-^ 33 ^)^.

There are various methodologies to communicate knowledge in an area. The development of online courses must consider the peculiarities of each area of study or the target audience for which it is developed^(^ 34 ^)^. In the case of nursing and health sciences in general, most MOOCs in health or specifically nursing-oriented are focused on the application of knowledge to this field of study or towards research or education in it, such as It can be seen in the courses found. Nursing is a profession that urgently involves problem solving, which means that the courses developed for this area must provide the student with the highest degree of knowledge and facilitate the development of skills that allow them to develop problems such as those they will face in the daily practice, hence the need for courses, in person or online, to evaluate the best strategies to provide this training to the nursing professional, and that it be in accordance with the required training standards^23^^,^ 34 ^)^.

This study sought to know the current state of supply of MOOCs in health and nursing as an opportunity for the continuing education of the professional nurse in Peru. It should be noted that MOOCs are launched according to the own considerations of each platform or of the institution developed for the course, the reported course offer responds to a historical one, being that some of the courses found may be available unlimitedly, others be offered during periods of the year and others are no longer available. Despite this limitation, the findings would indicate that there is an academic offer of MOOCs in health and nursing useful as a tool for online training of the nursing professional in Peru.

## Conclusion

There is a wide range of MOOCs available on online learning platforms in the health field, with courses specifically focused on nursing. The MOOCs offer includes courses in all areas of nursing considered by the CEP (professional care; public health, family and community health; teaching and research; organizational and management; and professional development). Most MOOCs are conducted by Anglo-Saxon institutions and are offered in the English language, so increasing the language options and transcripts in different languages for the audiovisual content of the MOOCS would be useful to encourage the participation of nursing professionals who do not have English as a mother tongue, in addition to promoting the development of courses by local institutions that, in addition to making MOOCs accessible in relation to the language, prepare courses that respond to the demand for training of the Peruvian nursing professional.
